# *In Utero* and Early-Life Exposure to Ambient Air Toxics and Childhood Brain Tumors: A Population-Based Case–Control Study in California, USA

**DOI:** 10.1289/ehp.1408582

**Published:** 2015-10-27

**Authors:** Ondine S. von Ehrenstein, Julia E. Heck, Andrew S. Park, Myles Cockburn, Loraine Escobedo, Beate Ritz

**Affiliations:** 1 Department of Community Health Sciences, and; 2 Department of Epidemiology, Fielding School of Public Health, University of California, Los Angeles, Los Angeles, California, USA; 3 Department of Preventive Medicine, Keck School of Medicine, University of Southern California, Los Angeles, California, USA

## Abstract

**Background:**

Little is known about the influence of environmental factors on the etiology of childhood brain tumors.

**Objectives:**

We examined risks for brain tumors in children after prenatal and infant exposure to monitored ambient air toxics.

**Methods:**

We ascertained all cases of medulloblastoma, central nervous system primitive neuroectodermal tumor (PNET), and astrocytoma before 6 years of age diagnosed in 1990–2007 from the California Cancer Registry and selected controls randomly from birth rolls matched by birth year. Exposures to air toxics during pregnancy/infancy for 43 PNET, 34 medulloblastoma, and 106 astrocytoma cases and 30,569 controls living within 5 mi of a monitor were determined. With factor analysis we assessed the correlational structures of 26 probable carcinogenic toxics, and estimated odds ratios by brain tumor type in logistic regression models.

**Results:**

PNETs (≤ 38 cases) were positively associated with interquartile range (IQR) increases in prenatal exposure to acetaldehyde [odds ratio (OR) = 2.30; 95% CI: 1.44, 3.67], 1,3-butadiene (OR = 2.23; 95% CI: 1.28, 3.88), benzene, and toluene; and with IQR increases in exposure during the first year of life to ortho-dichlorobenzene (OR = 3.27; 95% CI: 1.17, 9.14), 1,3-butadiene (OR = 3.15; 95% CI: 1.57, 6.32), and benzene. All exposures except ortho-dichlorobenzene loaded on the same factor. Medulloblastoma (≤ 30 cases) was associated with prenatal exposure to polycyclic aromatic hydrocarbons (PAHs combined: OR = 1.44; 95% CI: 1.15, 1.80). Exposures to lead and some PAHs during the first year of life were positively associated with astrocytoma, but the confidence intervals included the null value (e.g., for lead, OR = 1.40; 95% CI: 0.97, 2.03).

**Conclusions:**

Our data suggest that in utero and infancy exposures to air toxics generated by industrial and road traffic sources may increase the risk of PNET and medulloblastoma, with limited support for increased risks for astrocytoma in children up to age 6.

**Citation:**

von Ehrenstein OS, Heck JE, Park AS, Cockburn M, Escobedo L, Ritz B. 2016. In Utero and early-life exposure to ambient air toxics and childhood brain tumors: a population-based case–control study in California, USA. Environ Health Perspect 124:1093–1099; http://dx.doi.org/10.1289/ehp.1408582

## Introduction

Brain tumors are the most frequent solid tumors in children and the most common cause of childhood cancer deaths (Baldwin and Preston-Martin 2004). Among infants up to 36 months of age, the usually fast growing embryonal tumors medulloblastoma and central nervous system (CNS) primitive neuroectodermal tumor (PNET) are the most frequent brain neoplasms; among all children up to age 15 years, astrocytoma is the most common form of glioma (McKean-Cowdin et al. 2013) and the most common brain tumor subtype overall (Gurney 1999). Medulloblastoma is believed to arise from the precursor cells of the external granule layer of the developing cerebellum. PNET forms in the cerebrum and is composed of poorly differentiated neuroepithelial cells (MacDonald 2008). Medulloblastoma/PNET incidence is highest in infancy, declines slowly until age 5 years with a steep decline thereafter, whereas astrocytoma is reported to peak twice, at ages 5 and 13 years (Gurney 1999; McKean-Cowdin et al. 2013).

Although several genetic syndromes are associated with an increased risk for brain tumors, these syndromes are thought to account for < 5% of all cases (Baldwin and Preston-Martin 2004). Non-genetic risk factors still remain largely unknown. Although environmental influences are thought to play a key role in the development of childhood brain tumors, beyond high doses of ionizing radiation (IARC 2012), no environmental factor is an established risk factor. Suspected environmental factors include exposure to pesticides (Greenop et al. 2013; Searles Nielsen et al. 2010), parental occupational exposures (Cordier et al. 2001, 2004), paternal hobbies (Rosso et al. 2008), and maternal cured meat consumption and other dietary factors (Bunin et al. 2006; Searles Nielsen et al. 2011). Several studies have examined effects of maternal and paternal smoking, but findings are equivocal (Boffetta et al. 2000; Brooks et al. 2004; Milne et al. 2013).

Air toxics are defined by the U.S. Environmental Protection Agency (EPA) as pollutants that may cause serious health effects or adverse environmental and ecological effects, and are also known as hazardous air pollutants (HAPs). Many of these are common in urban air mixtures [e.g., polycyclic aromatic hydrocarbons (PAHs) or organic solvents] and are suspected or known carcinogens (IARC 2013), and have also been found to have adverse effects on the developing CNS (Calderón-Garcidueñas et al. 2008; Levesque et al. 2011). One previous study relied on modeled annual average HAPs and reported little association for gliomas (Reynolds et al. 2003).

To the best of our knowledge, no study to date has investigated perinatal exposure to monitored air toxics and specific subtypes of childhood brain tumors. Here we report on a California state-wide case–control study of childhood brain tumors and prenatal and infant exposure to monitored ambient air toxics, including PAHs, aromatic and chlorinated solvents, other volatile organic compounds, and several metals.

## Methods

### Study Design and Population

We ascertained all cases of medulloblastoma [*International Classification of Disease Oncology* (ICD-O) code 9470], PNET (ICD-O code 9473), and astrocytoma [*International Classification of Childhood Cancer, version 3* (ICCC-3) code 032] before age 6 years diagnosed in 1990–2007, from the California Cancer Registry (http://www.ccrcal.org). The overall study design has been described elsewhere (Heck et al. 2013a). In brief, we attempted to match all cancer cases to a California birth certificate (received from the Office of Vital Records, California Department of Public Health) using first and last names and dates of birth (89% matching rate). Controls without a cancer diagnosis before age 6 years were randomly selected from California birth rolls and frequency matched (20:1) by year of birth to all childhood cancer cases for the same time period. Date of birth and gestational age of each child were retrieved from birth certificates. From the entire cohort, 74 cases and 12,035 controls had missing gestational age and were excluded.

Human subjects research required for this study was approved by the institutional review boards of the University of California, Los Angeles, and the California Health and Human Services Agency; informed consent was waived because there was no contact with study subjects. Confidentiality was maintained by using only de-identified data in the analyses.

### Exposure Assessment

Residential addresses, as listed on the birth certificate, were geocoded using our open-source geocoder with manual correction of unmatched addresses (Goldberg et al. 2008) and used to classify exposure throughout pregnancy and during the first year of life. Exact home addresses were recorded on electronic birth certificates from 1998; before 1998, only ZIP codes were available, and we geocoded the ZIP code centroid for those children. The California Air Resources Board (CARB) has maintained an air toxics monitoring network since 1990, which collects 24-hr integrated samples of ambient air concentrations every 12 days. The 31 monitors (5-mi radius) were located across the state, positioned primarily near heavily trafficked highways, in industrial or in agriculturally intense regions at locations selected to be representative for the area (for map, see Cox et al. 2010). Using latitude/longitude locations provided by CARB, we determined the distance from each monitor to each home or ZIP code centroid, and participants were assigned pollutant values based upon the measurements taken at the nearest monitor. Based on categorization as “established, possible, or probable carcinogens” by the IARC (2013), we identified an initial set of 42 substances. For each toxic, we included children who had at least one reading for each full month of pregnancy and, because the last month of pregnancy rarely is exactly 1 month in length, with at least one reading within the last 30 days of pregnancy. We included in the analysis all subjects with geocoded addresses within < 5 mi from a CARB air toxics station to balance exposure misclassification with increasing distance from a station against sample size limitations. We further restricted the sample to children with gestational ages and birth weight considered viable (146–323 days, 500–6,800 g), and removed 719 controls because of death before age 6 years by matching to California death records. This resulted in 43 PNETs, 34 medulloblastomas, 106 astrocytomas, and 30,569 controls in the final sample (actual numbers of cases included in analyses varied and were less, due to missing information on exposure or covariates). For each pollutant, we included only children in the analysis who had at least one reading for each full month within the time period of interest. We included substances for which a minimum of 20 cases for each brain cancer type had values for the entire pregnancy average (i.e., at least one measurement for each month of pregnancy) assigned at 5 mi, resulting in 26 substances considered herein. Cases diagnosed during the first year of life were excluded from first year models. Time-specific exposure averages were generated based on birth date and gestational age as retrieved from birth certificates; we determined averages for each trimester, the entire pregnancy period, and the first year of life.

### Statistical Analyses

We employed Pearson’s correlation coefficients to examine collinearity across pollutants and pregnancy periods. We used factor analysis (varimax rotation) to create a correlation matrix for all 26 included exposures. This matrix helped us identify patterns of covariation of pollutants in our data that might represent common sources such as road traffic, or indicate mixtures of toxics in ambient air potentially acting together to increase cancer risks. Substances loading on the same factor might also be proxies for each other because of their high correlation. Whenever several agents loading on the same factor show similar and consistent results for the association with brain cancer, we believe that it supports the argument that either the whole mixture or at least a component of this mixture increases cancer risk. Unconditional logistic regression was used to estimate odds ratios (ORs) per interquartile-range (IQR) increase in pregnancy exposures for each toxicant during each trimester, the entire pregnancy, and the first 12 months of life. Selection of potential confounding variables was based upon previous knowledge (Baldwin and Preston-Martin 2004; McKean-Cowdin et al. 2013) as well as our own previous examination of demographic and perinatal factors related to cancer status in our data (data not shown). We adjusted all models for birth year (matching variable), and further adjusted models for maternal age, race/ethnicity, place of birth (United States vs. non–United States), and education (definitions as shown in Table 1). Additional adjustment for type of insurance [a measure of socioeconomic status (SES) in our population] (Ritz et al. 2007), an index variable of SES based on block group–level census data related to income, education, and occupation (Yost et al. 2001), rural/urban place of residence [based on U.S. Census 2000 data (U.S. Census Bureau 2013)], parity (primipara vs. one or more previous births), offspring sex, and preterm birth (< 37 weeks vs. ≥ 37 weeks) did not change the estimates of interest > 3% (data not shown) and thus were not retained in final models.

**Table 1:** Characteristics of the population for brain cancer cases and noncases residing within 5 mi of air monitoring stations at birth, California, birth years 1990–2007 [*n* (%)].

Characteristic	PNET (*n* = 43)	Medulloblastoma (*n* = 34)	Astrocytoma (*n* = 106)	Controls (*n* = 30,569)
Mother’s race/ethnicity
Non-Hispanic white	14 (32.6)	13 (38.2)	47 (44.3)	7,728 (25.3)
Hispanic of any race	20 (46.5)	14 (41.2)	40 (37.7)	16,169 (52.9)
Other/not specified	9 (20.9)	7 (20.6)	19 (17.9)	6,672 (21.8)
Mother’s age (years)
< 20	4 (9.3)	6 (17.7)	14 (13.2)	3,591 (11.8)
20–24	11 (25.6)	9 (26.5)	18 (17.0)	7,616 (24.9)
25–29	16 (37.2)	10 (29.4)	27 (25.5)	8,301 (27.2)
30–35	7 (16.3)	8 (23.5)	35 (33.0)	6,913 (22.6)
≥ 35	5 (11.6)	1 (2.9)	12 (11.3)	4,146 (13.6)
Missing	0	0	0	2 (0.01)
Source of payment for prenatal care
Public (Medi-Cal)	24 (55.8)	14 (41.2)	50 (47.2)	16,624 (54.4)
Private	19 (44.2)	19 (55.9)	56 (52.8)	13,671 (44.7)
Missing	0	1 (2.9)	0	274 (0.9)
Maternal education (years)
≤ 8	8 (18.6)	0 (0)	12 (11.3)	4,746 (15.5)
9–11	5 (11.6)	7 (20.6)	13 (12.3)	6,315 (20.7)
12	14 (32.6)	11 (32.4)	30 (28.3)	8,432 (27.6)
13–15	9 (20.9)	8 (23.5)	29 (27.4)	5,656 (18.5)
≥ 16	7 (16.3)	8 (23.5)	22 (20.8)	5,101 (16.7)
Missing	0	0	0	319 (1.0)
Urban^*a*^
Yes	39 (90.7)	33 (97.1)	100 (94.3)	28,807 (94.2)
No	4 (9.3)	1 (2.9)	6 (5.7)	1,762 (5.8)
USA born
Yes	19 (44.2)	23 (67.7)	63 (59.4)	14,388 (47.1)
No	24 (55.8)	11 (32.4)	43 (40.6)	16,181 (52.9)
Child sex
Male	27 (62.8)	21 (61.8)	50 (47.2)	15,499 (50.7)
Female	16 (37.2)	13 (38.2)	56 (52.8)	15,070 (49.3)
Parity
0	20 (46.5)	14 (41.2)	45 (42.5)	12,249 (40.1)
≥ 1	23 (53.5)	20 (58.8)	61 (57.6)	18,315 (59.9)
Missing	0	0	0	5 (0.01)
Preterm birth
Preterm	6 (14.0)	4 (11.8)	12 (11.3)	3,296 (10.8)
Term	37 (86.1)	30 (88.2)	94 (88.7)	27,273 (89.2)
Census-based SES^*b*^
1	15 (34.9)	7 (20.6)	26 (24.5)	9,483 (31.0)
2	13 (30.2)	8 (23.5)	25 (23.6)	7,397 (24.2)
3	6 (14.0)	6 (17.7)	22 (20.8)	5,476 (17.9)
4	8 (18.6)	8 (23.5)	22 (20.8)	5,407 (17.7)
5	1 (2.3)	5 (14.7)	11 (10.4)	2,806 (9.2)
Differences to 100% due to rounding; data is retrieved from birth certificates unless otherwise indicated. ^***a***^Urban/rural data based on census tract 2000 data (U.S. Census Bureau 2013). ^***b***^Census-based block-group level SES indicator variable,1 = lowest SES, 5 = highest SES.				

We present complete case analyses. Associations were evaluated based on the magnitudes of ORs and the width and position of the 95% confidence interval (CI) in relation to the null value. We have chosen not to adjust for multiple comparisons, based in part on the fact that all considered substances were selected *a priori* based on their classification as carcinogens by IARC (2013). Thus, the models presented equal the number of comparisons we conducted. Additionally, we also conducted sensitivity analyses (adding additional variables to the models mentioned above, restricting for term birth). All analyses were done with SAS version 9.3 (SAS Institute Inc., Cary, NC).

## Results

Most demographic characteristics were similar among cases and controls, except that more PNET and medulloblastoma case children were boys (Table 1), which is consistent with national data (Ostrom et al. 2015). Mean (± SD) age in years at diagnosis for PNET, medulloblastoma, and astrocytoma was 2.5 ± 1.6, 2.0 ± 1.5, and 2.5 ± 1.8, respectively. The means, standard deviation, and factor loadings (> 0.6) of the air toxics are displayed in Table S1.

Risks for PNET increased in relation to IQR increases during pregnancy and first year of life for substances loading on factor 1, particularly to aromatic solvents [benzene, toluene, ethyl-benzene, and ortho-xylene (collectively referred to as BTEX), and 1,3-butadiene] and the chlorinated solvents perchloroethylene and trichloroethylene, as well as to acetaldehyde and selenium. PNET risk also increased related to pregnancy and infant exposure to formaldehyde and chloroform, both loading on factor 2 (Table 2). Associations with prenatal exposures to benzene, ethyl-benzene, ortho-xylene, butadiene, and selenium were strongest for exposures during the first two trimesters, whereas associations with toluene, acetaldehyde, perchloroethylene, and trichloroethylene were similar for exposures during all trimesters (see Table S2). PNET was positively associated with hexavalent chromium exposure during the second trimester (OR = 1.10; 95% CI: 0.99, 1.22), and with styrene exposure in the first and second trimesters (OR = 1.31; 95% CI: 0.99, 1.73 and OR = 1.24; 95% CI: 0.94, 1.64, respectively). PNET was positively associated with lead exposure during all trimesters (e.g., OR = 1.23; 95% CI: 0.85, 1.79 for an IQR increase in lead during the first trimester). ortho-Dichlorobenzene (factor 2) was associated with PNET based on postnatal exposure, and there was an increased risk suggested for exposure during the first two trimesters [trimester 1: OR = 1.23 (95% CI: 0.76, 2.01); trimester 2: OR = 1.51 (0.98, 2.34)] (see Table S2).

**Table 2:** Adjusted*^a^* odds ratios for *in utero* and first-year-of-life exposure to air toxics and primitive neuroectodermal tumors in children by age 6 years residing within 5 mi of monitoring stations at birth, California, birth years 1990–2007.

Air toxic	Prenatal			1st year of life	
	IQR	Case/control (*n*)	OR^*a*^ (95% CI)	Case/control (*n*)	OR^*a*^ (95% CI)
Factor 1
Aromatic solvents
Toluene (ppbV)	2.196	37/24,149	2.14 (1.38, 3.32)	30/22,528	2.19 (1.32, 3.65)
ortho-Xylene (ppbV)	0.388	37/24,033	1.83 (1.22, 2.74)	30/22,376	1.88 (1.15, 3.07)
Ethyl benzene (ppbV)	0.178	35/23,267	1.59 (1.13, 2.26)	28/21,648	1.75 (1.12, 2.73)
1,3-Butadiene (ppbV)	0.257	38/27,189	2.23 (1.28, 3.88)	31/25,688	3.15 (1.57, 6.32)
Benzene (ppbV)	1.216	38/27,199	2.14 (1.12, 4.06)	31/25,698	2.42 (1.09, 5.37)
Chlorinated solvents
Perchloroethylene (ppbV)	0.231	36/25,061	1.52 (1.13, 2.04)	28/22,657	1.68 (1.14, 2.49)
Tricloroethylene (ppbV)	0.054	36/25,168	1.19 (1.07, 1.32)	29/23,343	1.22 (1.05, 1.42)
Methylene chloride (ppbV)	0.453	34/25,412	1.14 (0.93, 1.40)	29/23,758	1.13 (0.85, 1.49)
Other
Hexavalent chromium (ng/m^3^)	0.134	26/16,944	1.23 (0.89, 1.68)	21/14,038	1.11 (0.68, 1.82)
Lead (ng/m^3^)	20.048	26/19,765	1.38 (0.85, 2.25)	20/16,680	1.34 (0.74, 2.44)
Styrene (ppbV)	0.137	29/20,001	1.31 (0.88, 1.94)	21/17,132	1.27 (0.72, 2.25)
Acetaldehyde (ppbV)	0.900	34/25,361	2.30 (1.44, 3.67)	29/23,585	2.08 (1.25, 3.46)
Selenium (ng/m^3^)	0.732	25/18,999	1.58 (1.15, 2.17)	19/15,850	1.87 (1.20, 2.92)
Factor 2
PAHs^*b*^ (ng/m^3^)	1.049	29/21,368	1.06 (0.73, 1.55)	20/17,862	1.03 (0.53, 2.01)
Benzo[*k*]fluoranthene	0.077	30/22,416	0.96 (0.68, 1.37)	21/18,834	1.14 (0.73, 1.77)
Benzo[*b*]fluoranthene	0.192	30/22,416	1.00 (0.71, 1.42)	21/18,834	1.11 (0.68, 1.84)
Indeno[1,2,3-*cd*]pyrene	0.233	29/21,368	1.04 (0.71, 1.52)	20/17,862	1.03 (0.51, 2.05)
Benzo[*a*]pyrene	0.157	30/22,416	0.93 (0.66, 1.31)	21/18,834	1.09 (0.72, 1.65)
Dibenz[*a,h*]anthracene	0.015	29/21,368	0.81 (0.56, 1.19)	20/17,862	0.83 (0.48, 1.45)
Benzo[*g,h,i*]perylene	0.448	29/21,368	1.58 (0.95, 2.63)	20/17,862	1.73 (0.73, 4.10)
Other (ppbV)
Chloroform	0.017	37/25,534	1.48 (1.07, 2.05)	30/23,792	1.50 (1.01, 2.21)
*ortho*-Dichlorobenzene	0.076	32/21,053	1.51 (0.75, 3.04)	23/18,040	3.27 (1.17, 9.14)
*para*-Dichloro-benzene	0.039	32/21,121	1.25 (0.96, 1.63)	23/18,093	1.12 (0.72, 1.73)
Formaldehyde	1.334	34/25,361	1.32 (1.00, 1.75)	29/23,585	1.68 (1.16, 2.43)
Not loading (ng/m^3^)
Chromium	3.206	26/19,867	1.24 (0.86, 1.77)	20/16,703	1.21 (0.76, 1.91)
Nickel	3.196	26/19,889	1.17 (0.67, 2.05)	20/16,703	1.20 (0.61, 2.35)
^***a***^Adjusted for birth year, maternal race/ethnicity, maternal age and education, place of birth mother (USA vs. non-USA). ^***b***^PAHs include the sum of average concentrations of six hydrocarbons: benzo[*a*]pyrene, benzo[*b*]fluoranthene, benzo[*g,h,i*]perylene, benzo[*k*]fluoranthene, dibenz[*a,h*]anthracene, and indeno[1,2,3-*c,d*]pyrene.					

Medulloblastoma was positively associated with prenatal and first-year-of-life exposure to PAHs (Table 3). For example, ORs for IQR increases in summed PAHs *in utero* and during the first year of life were 1.44 (95% CI: 1.15, 1.80) and 1.48 (95% CI: 0.85, 2.57) based on 27 and 18 exposed cases, respectively. When evaluated by trimester, associations with PAHs were closer to the null but positive for the first and second trimesters [OR = 1.13 (95% CI: 1.01, 1.26) and OR = 1.10 (95% CI: 0.99, 1.22), respectively, for summed PAHs] (see Table S3). ORs were < 1 for several factor 1 substances, and about a quarter of the factor 2 substances; however, the 95% CIs were very wide, indicating low precision, and included the null value.

**Table 3:** Adjusted*^a^* odds ratios for *in utero* and first-year-of-life exposure to air toxics and medulloblastoma in children by age 6 years residing within 5 mi of monitoring stations at birth, California, birth years 1990–2007.

Air toxic	Prenatal			1st Year of life	
	IQR	Case/control (*n*)	OR^*a*^ (95% CI)	Case/control (*n*)	OR^*a*^ (95% CI)
Factor 1
Aromatic solvents
Toluene (ppbV)	2.196	27/24,149	0.66 (0.33, 1.33)	20/22,528	0.90 (0.40, 2.00)
ortho-Xylene (ppbV)	0.388	27/24,033	0.78 (0.42, 1.46)	20/22,376	0.78 (0.35, 1.71)
Ethyl benzene (ppbV)	0.178	24/23,267	0.50 (0.24, 1.03)	18/21,648	0.73 (0.34, 1.60)
1,3-Butadiene (ppbV)	0.257	30/27,189	0.76 (0.36, 1.62)	21/25,688	0.67 (0.24, 1.90)
Benzene (ppbV)	1.216	30/27,199	0.82 (0.36, 1.87)	21/25,698	0.54 (0.16, 1.81)
Chlorinated solvents
Perchloroethylene (ppbV)	0.231	28/25,061	0.48 (0.22, 1.03)	20/22,657	0.72 (0.33, 1.59)
Tricloroethylene (ppbV)	0.054	28/25,168	0.93 (0.71, 1.20)	20/23,343	1.04 (0.78, 1.38)
Methylene chloride (ppbV)	0.453	28/25,412	0.63 (0.36, 1.12)	20/23,758	0.65 (0.32, 1.35)
Other
Hexavalent chromium (ng/m^3^)	0.134	20/16,944	0.33 (0.09, 1.18)	18/14,038	0.60 (0.18, 2.03)
Lead (ng/m^3^)	20.048	21/19,765	0.95 (0.49, 1.84)	17/16,680	1.00 (0.42, 2.40)
Styrene (ppbV)	0.137	25/20,001	0.95 (0.56, 1.62)	14/17,132	0.96 (0.43, 2.14)
Acetaldehyde (ppbV)	0.900	27/25,361	0.84 (0.46, 1.53)	21/23,585	0.82 (0.41, 1.65)
Selenium (ng/m^3^)	0.732	20/18,999	1.05 (0.63, 1.76)	17/15,850	1.20 (0.66, 2.19)
Factor 2
PAHs^*b*^ (ng/m^3^)	1.049	27/21,368	1.44 (1.15, 1.80)	18/17,862	1.48 (0.85, 2.57)
Benzo[*k*]fluoranthene	0.077	28/22,416	1.28 (1.07, 1.52)	18/18,834	1.32 (0.89, 1.98)
Benzo[*b*]fluoranthene	0.192	28/22,416	1.33 (1.10, 1.61)	18/18,834	1.36 (0.88, 2.10)
Indeno[1,2,3-*cd*]pyrene	0.233	27/21,368	1.38 (1.13, 1.69)	18/17,862	1.61 (0.91, 2.83)
Benzo[*a*]pyrene	0.157	28/22,416	1.25 (1.06, 1.46)	18/18,834	1.30 (0.92, 1.86)
Dibenz[*a,h*]anthracene	0.015	27/21,368	1.07 (1.01, 1.14)	18/17,862	1.20 (0.84, 1.72)
Benzo[*g,h,i*]perylene	0.448	27/21,368	1.94 (1.20, 3.14)	18/17,862	1.50 (0.56, 3.99)
Other (ppbV)
Chloroform	0.017	28/25,534	0.77 (0.45, 1.31)	20/23,792	0.75 (0.39, 1.44)
*ortho*-Dichlorobenzene	0.076	23/21,053	0.59 (0.22, 1.64)	14/18,040	1.08 (0.23, 5.15)
*para*-Dichloro-benzene	0.039	23/21,121	0.99 (0.66, 1.48)	14/18,093	1.35 (0.81, 2.23)
Formaldehyde	1.334	27/25,361	0.79 (0.46, 1.33)	21/23,585	0.84 (0.47, 1.48)
Not loading (ng/m^3^)
Chromium	3.206	21/19,867	0.82 (0.43, 1.55)	17/16,703	0.82 (0.40, 1.70)
Nickel	3.196	21/19,889	0.69 (0.34, 1.44)	17/16,703	0.62 (0.26, 1.52)
^***a***^Adjusted for birth year, maternal race/ethnicity, maternal age and education, place of birth mother (USA vs. non-USA). ^***b***^PAHs include the sum of average concentrations of six hydrocarbons: benzo[*a*]pyrene, benzo[*b*]fluoranthene, benzo[*g,h,i*]perylene, benzo[*k*]fluoranthene, dibenz[*a,h*]anthracene, and indeno[1,2,3-*c,d*]pyrene.					

For astrocytoma, relatively small risk increases were suggested for exposures during the first year of life for lead (OR = 1.40; 95% CI: 0.97, 2.03), some PAHs (e.g., benzo[*k*]fluoranthene OR = 1.20; 95% CI: 0.95, 1.51) and trichloroethylene (OR = 1.10; 95% CI: 0.97, 1.24) (Table 4). No associations were suggested for prenatal exposure and astrocytoma (Table 4).

**Table 4:** Adjusted*^a^* odds ratios for *in utero* and first-year-of-life exposure to air toxics and astrocytoma in children by age 6 years residing within 5 mi of monitoring stations at birth, 1990–2007, California.

Air toxic	Prenatal			1st Year of life	
	IQR	Case/Control (*n*)	OR^*a*^ 95% CI	Case/Control (*n*)	OR^*a*^ 95% CI
Factor 1
Aromatic solvents
Toluene (ppbV)	2.196	82/24,149	0.89 (0.62, 1.29)	67/22,528	0.95 (0.62, 1.47)
ortho-Xylene (ppbV)	0.388	81/24,033	0.87 (0.61, 1.23)	66/22,376	0.87 (0.57, 1.33)
Ethyl benzene (ppbV)	0.178	83/23,267	1.05 (0.80, 1.39)	68/21,648	1.10 (0.78, 1.56)
1,3-Butadiene (ppbV)	0.257	100/27,189	0.93 (0.63, 1.37)	85/25,688	1.05 (0.66, 1.66)
Benzene (ppbV)	1.216	100/27,199	0.83 (0.53, 1.29)	85/25,698	0.87 (0.51, 1.50)
Chlorinated solvents
Perchloroethylene (ppbV)	0.231	89/25,061	0.92 (0.69, 1.23)	69/22,657	1.08 (0.78, 1.51)
Tricloroethylene (ppbV)	0.054	89/25,168	1.05 (0.95, 1.16)	71/23,343	1.10 (0.97, 1.24)
Methylene chloride (ppbV)	0.453	92/25,412	0.93 (0.77, 1.14)	76/23,758	0.99 (0.80, 1.24)
Other
Hexavalent chromium (ng/m^3^)	0.134	64/16,944	0.54 (0.28, 1.01)	43/14,038	0.87 (0.50, 1.52)
Lead (ng/m^3^)	20.048	74/19,765	1.22 (0.89, 1.67)	61/16,680	1.40 (0.97, 2.03)
Styrene (ppbV)	0.137	67/20,001	0.73 (0.51, 1.04)	47/17,132	0.70 (0.42, 1.17)
Acetaldehyde (ppbV)	0.900	92/25,361	0.96 (0.70, 1.30)	77/23,585	0.90 (0.64, 1.26)
Selenium (ng/m^3^)	0.732	69/18,999	1.05 (0.80, 1.37)	56/15,850	0.88 (0.60, 1.29)
Factor 2
PAHs^*b*^ (ng/m^3^)	1.049	77/21,368	1.06 (0.85, 1.33)	59/17,862	1.17 (0.81, 1.69)
Benzo[*k*]fluoranthene	0.077	83/22,416	1.05 (0.90, 1.24)	65/18,834	1.20 (0.95, 1.51)
Benzo[*b*]fluoranthene	0.192	83/22,416	1.06 (0.89, 1.26)	65/18,834	1.19 (0.92, 1.54)
Indeno[1,2,3-*cd*]pyrene	0.233	77/21,368	1.09 (0.89, 1.34)	59/17,862	1.19 (0.81, 1.73)
Benzo[*a*]pyrene	0.157	83/22,416	1.04 (0.90, 1.20)	65/18,834	1.16 (0.94, 1.44)
Dibenz[*a,h*]anthracene	0.015	77/21,368	1.03 (0.96, 1.10)	59/17,862	1.08 (0.86, 1.37)
Benzo[*g,h,i*]perylene	0.448	77/21,368	0.96 (0.67, 1.37)	59/17,862	1.05 (0.60, 1.83)
Other (ppbV)
Chloroform	0.017	93/25,534	0.91 (0.69, 1.19)	77/23,792	1.18 (0.90, 1.55)
*ortho*-Dichlorobenzene	0.076	74/21,053	1.19 (0.72, 1.96)	54/18,040	1.42 (0.64, 3.16)
*para*-Dichloro-benzene	0.039	74/21,121	1.11 (0.90, 1.35)	54/18,093	1.05 (0.75, 1.46)
Formaldehyde	1.334	92/25,361	1.20 (0.96, 1.49)	77/23,585	1.11 (0.85, 1.43)
Not loading (ng/m^3^)
Chromium	3.206	74/19,867	1.01 (0.77, 1.33)	61/16,703	0.98 (0.70, 1.35)
Nickel	3.196	74/19,889	0.83 (0.58, 1.20)	61/16,703	0.83 (0.54, 1.29)
^***a***^Adjusted for birth year, maternal race/ethnicity, maternal age and education, place of birth mother (USA vs. non-USA). ^***b***^PAH: Includes sum of average concentrations of six hydrocarbons: benzo[*a*]pyrene, benzo[*b*]fluoranthene, benzo[*g,h,i*]perylene, benzo[*k*]fluoranthene, dibenz[*a,h*]anthracene, and indeno[1,2,3-*c,d*]pyrene.					

For PNET and medulloblastoma, associations were generally stronger (OR further from the null) with exposure during the entire pregnancy than associations with exposure according to trimester of pregnancy (Tables 2 and 3; see also Tables S2 and S3).

## Discussion

In this first large population-based childhood brain cancer study investigating ambient air toxics measured at community-based monitoring stations, we found increased risks for embryonal brain tumors in young children related to estimated exposure during fetal and first-year-of-life brain development. PNET risks were associated with pre- and postnatal exposure to several correlated toxics (butadiene, BTEX, selenium, acetaldehyde, perchlorethylene, trichloroethylene, chloroform), and to first-year exposure of ortho-dichlorobenzene. Medulloblastoma risks were associated with higher prenatal PAH exposures. For astrocytoma we estimated imprecise increases in risk (with the OR around 20% > 1 and the lower 95% CI close to excluding the null) related to exposures to lead and some PAHs in the first year of life. As astrocytoma continues to be diagnosed through later childhood, we may not be capturing the most relevant time period for this tumor in our study of children < 6 years of age.

Very little research on environmental contributions to the etiology of childhood brain cancer has been published, and to our knowledge no prior study has examined pre- and postnatal exposure to monitored concentrations of common ambient air toxics. One small case-only study (*n* = 98) considered concentrations of annual chlorinated solvents modeled at the census tract level and reported an interaction between high trichloroethylene and *OGG1* rs293795 genotype and childhood medulloblastoma/PNET (Lupo et al. 2012); the case-only study design did not allow the researchers to estimate marginal effects for the chlorinated solvents. The only other study to date we are aware of that considered a range of HAPs and childhood cancers included brain tumors diagnosed in children up to age 15 years (1988–1994). This study relied on county-level modeled annual averages from 1990 using the U.S. EPA HAP emissions model. The authors created different exposure scores combining 25 frequent HAPs, and combining point versus mobile sources of emissions, and they considered cancers at all sites, and differentiated only between leukemias and gliomas. Positive associations were reported for leukemias (OR = 1.32; 95% CI: 1.11, 1.57), and for gliomas the OR was 1.19 (95% CI: 0.96, 1.46); embryonal brain tumors were not considered separately (Reynolds et al. 2003).

A few previous studies have investigated traffic-related exposure in relation to all childhood cancers, including brain tumors, and suggested increased risks for leukemia but found little indication of association for other tumors (Raaschou-Nielsen et al. 2001; Reynolds et al. 2002, 2004). One previous California study of all childhood cancers used density of roadways within 500 ft around the birth address as an indicator of traffic exposure and reported for “higher traffic density” an OR of 1.22 (95% CI: 0.87, 1.70) for combined CNS tumors (Reynolds et al. 2004). In our own recent study of all California childhood cancers assessing traffic related air pollution using CAlifornia LINE (CALINE4) Source Dispersion Modeling, we found associations between IQR increases in modeled carbon monoxide estimated using CALINE4 and acute lymphoblastic leukemia and retinoblastoma and estimated an OR of 1.10 (95% CI: 0.93, 1.31) for PNET (Heck et al. 2013c). Employing a land use regression (LUR) model to assess traffic related exposure in Los Angeles County only, we examined PNET and astrocytoma but found no associations (Ghosh et al. 2013). We reported earlier no more than moderate correlations (*r* = 0.2 to 0.5) between several air toxics including benzene and PAHs and LUR-based measures of nitrogen oxides exposure in Los Angeles County (Ghosh et al. 2012). This indicates that the LUR-based exposure markers may not be good indicators for these air toxics from industry and traffic sources in Los Angeles or for the mixture of air toxics across California, and may explain the differences in findings between the studies using LUR-based exposure estimates versus estimates based on air toxics monitoring data.

Our own recent exploratory study (using the same California study as for the brain cancer study) of air toxics and childhood neuroblastoma (*n* cases = 75, *n* controls = 14,602), an embryonal malignancy of the sympathetic nervous system, suggested slightly increased risks related to prenatal PAHs and carbon tetrachloride exposure (Heck et al. 2013b). We also found leukemia and retinoblastoma to be positively related to several toxics generated in fuel combustion and traffic, and to chloroform (Heck et al. 2014, 2015), which is in line with our present findings. Factors we identified based on substances with similar loadings may be representative of common or similar emission sources and their complex mixtures of air toxics. It is possible that the combined exposures rather than single substances contribute to CNS tumor risk. The exposures that were most strongly associated with PNET in the present analysis included several substances loading on factor 1 including acetaldehyde, butadiene, benzene, toluene, and related aromatic solvents, which are generated in fossil fuel burning with primary sources in California being fuel combustion, combustion processes in petroleum refining and oil and gas extraction, coke oven operations, and forest fires (Cox et al. 2010). Selenium additionally is emitted in the production and refining of copper (George 2003). The chlorinated solvents perchloroethylene and trichloroethylene are frequently used in the textile industry and in dry cleaning, whereas chloroform (factor 2) is frequently generated in wastewater treatment (U.S. Geological Survey 2015). Exposure during the first year of life to ortho-dichlorobenzene (factor 2), which is mainly generated in agricultural pesticide production, was positively associated with PNET. Because most of the substances associated with PNET were highly correlated, our ability to distinguish whether and which specific substances or the mixture of these established or probable carcinogens are responsible for the outcome is limited (Dominici et al. 2010). Future studies need to confirm the associations we reported here and rule out possible residual confounding and to be designed specifically for the purpose of disentangling whether specific toxics or the combination of several toxics increase childhood brain cancer risk.

There was also some suggestion of increased risks for astrocytoma and lead exposure in infancy. Preconceptional paternal occupational lead exposure was inversely associated with astrocytomas and embryonal tumors in a large UK study; however, maternal exposure was not assessed (Keegan et al. 2013). An adult brain cancer study found positive associations with lead (Rajaraman et al. 2006). Yet data on the risks for childhood brain tumors related to metals are sparse.

Medulloblastoma ORs increased moderately with prenatal exposure to PAHs, which are emitted through coal, wood or fuel burning, petroleum refining, coke production, and tobacco smoke. Little prior research on childhood brain tumors and PAH exposure exists. One study of cancers in children up to age 19 years in relation to maternal prenatal and paternal preconceptional occupational PAH exposure found slight increases related to the latter but not for maternal exposures. However, maternal exposure was rare, and no actual PAH measurements were undertaken (Cordier et al. 2004). The authors reported increased ORs for astroglial tumors (classified using ICD-O codes); we only estimated weak and imprecise associations with exposures during the first year of life for astrocytoma (classified according to ICCC-3). Cordier et al. (2004) also combined medulloblastoma/PNET into one group, further limiting our ability to compare these results to our study.

Several childhood brain tumor studies investigated parental occupational exposures involving exposures to some toxic air pollutants. Parental occupations related to vehicle exhaust, and maternal exposure to solvents and maternal employment in health care (Cordier et al. 1997), as well as in the textile industry, increased risk for PNET and other brain tumors (Cordier et al. 2001). Maternal prenatal and paternal periconceptional exposure to diesel exhaust were related to increased risks for all childhood brain tumors combined by age 5 years, in a case–control study (Peters et al. 2013). A United Kingdom–wide study of paternal occupations found no association with PAH exposure or occupations involving solvents and all CNS tumors combined; however, intrauterine exposure via maternal occupation was not considered (Keegan et al. 2013). Finally, several studies have examined effects of maternal and paternal smoking, but findings are equivocal with positive associations seen for paternal but not maternal smoking during or after pregnancy in an earlier meta-analysis (Boffetta et al. 2000). Positive associations between maternal prenatal smoking and child brain tumors were reported based on prospectively collected data in Sweden (Brooks et al. 2004). Recently, no overall association for parental prepregnancy or prenatal smoking was found; however, there was some indication of increased risks for diagnosis before age 2 years, but the number of young cases was small (Milne et al. 2013). Differential misreporting of prenatal smoking with parents of cases underreporting smoking, may have contributed to bias in retrospective studies (Czeizel et al. 2004). Diet may be another source of exposure to PAH (Ma and Harrad 2015); cured meat has been found to contain elevated concentrations of PAH as well as nitrosamines (Li et al. 2012; Ma and Harrad 2015), but studies examining associations between cured meat and childhood brain tumors are rare and the findings equivocal (Searles Nielsen et al. 2011).

Although we cannot disentangle estimated effects of single substances from those of mixtures because of the high correlation of many air toxics, the associations we see with PNET and medulloblastoma, respectively, for different groups of pollutants may suggest that these toxics potentially induce (tumor-specific) genetic alterations in key neurodevelopmental pathways. Medulloblastoma is thought to arise from genetic anomalies in developmental pathways required for the normal maturation of the cerebellar cortex (Hatten and Roussel 2011), including key developmental signaling pathways such as Notch, Wnt, and Hedgehog (Karamboulas and Ailles 2013). Less is known about pathophysiology for PNET, but gene expression data suggest that Wnt signaling pathway activation disrupts normal differentiation in the CNS (Rogers et al. 2013). Based on animal studies, the most striking finding in the induction of brain tumors is the much greater susceptibility of the fetal and neonatal nervous system to some carcinogens, compared with the nervous system in adults of the same species (Rice and Wilbourn 2000). Mechanistic studies are needed to elucidate whether the air toxics exposure related increased risks we observed for the embryonal brain tumors in fact relate to underlying carcinogenic and developmental mechanisms.

Our study has a number of limitations. We do not have data for similar types of exposures from other sources—for indoor air, occupation, smoking, or diet—that may contribute to exposure misclassification. However, for the higher-molecular-weight PAHs we examined in the present study, indoor measurements of PAHs are strongly correlated with outdoor concentrations (Naumova et al. 2002). Misclassification is also possible due to the reliance upon birth address used to assess exposures throughout pregnancy; 9% to 30% of families move residence during pregnancy, based on a systematic review including populations from several U.S. states and Europe (Bell and Belanger 2012). Moves occur mainly in the second trimester, which likely affects the accuracy of early-pregnancy exposures. However, most moves were found to be local (< 10 km) (Bell and Belanger 2012), which likely limits biases from misclassification for our exposure assessment based on a 5-mi radius. We do not have data on the percentage of children who moved during the first year of life in our study; however, geocoding methods were based on the place of birth for all children so misclassification is the same for cases and controls. We adjusted our models for important confounders and conducted sensitivity analyses adding additional variables such as parity, child sex, SES indicators, and rural/urban location, which did not change our estimates; yet residual confounding due to unmeasured factors is always possible. Although we relied on a large sample for a childhood cancer study, the rarity of childhood brain tumors combined with the limited number of air toxics monitoring stations resulted in small numbers of cases with exposure measurements and reduced statistical power. Comparing control subjects in the present study to controls in the parent study sample (born during the same time period) but not living within 5 mi of an air monitor, we saw that controls in the present study differed only slightly for most variables except that there were more Hispanics (52.9% vs. 44.4%), more children without private health insurance (54.4% vs. 49.3%), and fewer U.S.-born mothers (47.1% vs. 57.0%) in our sample. Furthermore, a higher percentage was urban (94.2% vs. 79.5), which reflects the fact that we have fewer air toxics monitors and lower population density in rural areas. Thus, we were unable to adequately estimate air toxics exposure and potential risks due to pesticide applications. Among controls missing versus not missing gestational age, fewer indicated private health insurance (35.1% vs. 44.7%) or Hispanic ethnicity (44.9% vs. 52.9%), and more were U.S. born (59.9% vs. 47.1%); other characteristics did not differ. One limitation inherent to the field of childhood brain tumor research is the small number for each tumor type. Strengths of our study include the population based design and record-based approach, which eliminates the drawbacks of recall bias as well as selection bias due to nonparticipation and the ability to differentiate between brain cancer subtypes.

In conclusion, our findings suggest increased risks for the highly malignant and difficult to treat embryonal CNS tumors PNET and medulloblastoma related to *in utero* and infancy exposure to air toxics emitted from industrial and road traffic sources at ambient concentrations occurring in communities in California.

## Supplemental Material

(272 KB) PDF

## References

[r1] Baldwin RT, Preston-Martin S (2004). Epidemiology of brain tumors in childhood—a review.. Toxicol Appl Pharmacol.

[r2] Bell ML, Belanger K (2012). Review of research on residential mobility during pregnancy: consequences for assessment of prenatal environmental exposures.. J Expo Sci Environ Epidemiol.

[r3] Boffetta P, Trédaniel J, Greco A (2000). Risk of childhood cancer and adult lung cancer after childhood exposure to passive smoke: a meta-analysis.. Environ Health Perspect.

[r4] Brooks DR, Mucci LA, Hatch EE, Cnattingius S (2004). Maternal smoking during pregnancy and risk of brain tumors in the offspring. A prospective study of 1.4 million Swedish births.. Cancer Causes Control.

[r5] Bunin GR, Gallagher PR, Rorke-Adams LB, Robison LL, Cnaan A (2006). Maternal supplement, micronutrient, and cured meat intake during pregnancy and risk of medulloblastoma during childhood: a children’s oncology group study.. Cancer Epidemiol Biomarkers Prev.

[r6] Calderón-Garcidueñas L, Mora-Tiscareño A, Ontiveros E, Gómez-Garza G, Barragán-Mejía G, Broadway J (2008). Air pollution, cognitive deficits and brain abnormalities: a pilot study with children and dogs.. Brain Cogn.

[r7] Cordier S, Lefeuvre B, Filippini G, Peris-Bonet R, Farinotti M, Lovicu G (1997). Parental occupation, occupational exposure to solvents and polycyclic aromatic hydrocarbons and risk of childhood brain tumors (Italy, France, Spain).. Cancer Causes Control.

[r8] Cordier S, Mandereau L, Preston-Martin S, Little J, Lubin F, Mueller B (2001). Parental occupations and childhood brain tumors: results of an international case–control study.. Cancer Causes Control.

[r9] Cordier S, Monfort C, Filippini G, Preston-Martin S, Lubin F, Mueller BA (2004). Parental exposure to polycyclic aromatic hydrocarbons and the risk of childhood brain tumors: the SEARCH International Childhood Brain Tumor Study.. Am J Epidemiol.

[r10] Cox P, Delao A, Komorniczak A, Weller R (2010). The 2009 California Almanac of Emissions and Air Quality—2009 Edition.. http://www.arb.ca.gov/aqd/almanac/almanac09/almanac2009all.pdf.

[r11] Czeizel AE, Petik D, Puho E (2004). Smoking and alcohol drinking during pregnancy. The reliability of retrospective maternal self-reported information.. Cent Eur J Public Health.

[r12] Dominici F, Peng RD, Barr CD, Bell ML (2010). Protecting human health from air pollution: shifting from a single-pollutant to a multipollutant approach.. Epidemiology.

[r13] George M (2003). Selenium and tellurium.. In: Metals and Minerals: U.S. Geological Survey Minerals Yearbook. United States Geological Survey.

[r14] Ghosh JK, Heck JE, Cockburn M, Su J, Jerrett M, Ritz B (2013). Prenatal exposure to traffic-related air pollution and risk of early childhood cancers.. Am J Epidemiol.

[r15] Ghosh JK, Wilhelm M, Su J, Goldberg D, Cockburn M, Jerrett M (2012). Assessing the influence of traffic-related air pollution on risk of term low birth weight on the basis of land-use-based regression models and measures of air toxics.. Am J Epidemiol.

[r16] Goldberg DW Wilson JP Knoblock CA Ritz B Cockburn MG 2008 An effective and efficient approach for manually improving geocoded data. Int J Health Geogr 7 60, doi:10.1186/1476-072X-7-60 19032791 PMC2612650

[r17] Greenop KR, Peters S, Bailey HD, Fritschi L, Attia J, Scott RJ (2013). Exposure to pesticides and the risk of childhood brain tumors.. Cancer Causes Control.

[r18] Gurney JG (1999). Topical topics: brain cancer incidence in children: time to look beyond the trends.. Med Pediatr Oncol.

[r19] Hatten ME, Roussel MF (2011). Development and cancer of the cerebellum.. Trends Neurosci.

[r20] Heck JE, Lombardi CA, Cockburn M, Meyers TJ, Wilhelm M, Ritz B (2013a). Epidemiology of rhabdoid tumors of early childhood.. Pediatr Blood Cancer.

[r21] Heck JE, Park AS, Qiu J, Cockburn M, Ritz B (2013b). An exploratory study of ambient air toxics exposure in pregnancy and the risk of neuroblastoma in offspring.. Environ Res.

[r22] Heck JE, Park AS, Qiu J, Cockburn M, Ritz B (2014). Risk of leukemia in relation to exposure to ambient air toxics in pregnancy and early childhood.. Int J Hyg Environ Health.

[r23] Heck JE, Park AS, Qiu J, Cockburn M, Ritz B (2015). Retinoblastoma and ambient exposure to air toxics in the perinatal period.. J Expo Sci Environ Epidemiol.

[r24] Heck JE Wu J Lombardi C Qiu J Meyers TJ Wilhelm M 2013c Childhood cancer and traffic-related air pollution exposure in pregnancy and early life. Environ Health Perspect 121 1385 1391, doi:10.1289/ehp.1306761 24021746 PMC3855517

[r25] IARC (International Agency for Research on Cancer) (2012). Radiation.. IARC Mongr Eval Carcinog Risk Hum 100D.

[r26] IARC (2013). Agents Classified by the *IARC Monographs*, 1–112.. http://monographs.iarc.fr/ENG/Classification/ClassificationsAlphaOrder.pdf.

[r27] Karamboulas C, Ailles L (2013). Developmental signaling pathways in cancer stem cells of solid tumors.. Biochim Biophys Acta.

[r28] Keegan TJ, Bunch KJ, Vincent TJ, King JC, O’Neill KA, Kendall GM (2013). Case-control study of paternal occupation and social class with risk of childhood central nervous system tumours in Great Britain, 1962–2006.. Br J Cancer.

[r29] Levesque S Taetzsch T Lull ME Kodavanti U Stadler K Wagner A 2011 Diesel exhaust activates and primes microglia: air pollution, neuroinflammation, and regulation of dopaminergic neurotoxicity. Environ Health Perspect 119 1149 1155, doi:10.1289/ehp.1002986 21561831 PMC3237351

[r30] Li L, Wang P, Xu X, Zhou G (2012). Influence of various cooking methods on the concentrations of volatile N-nitrosamines and biogenic amines in dry-cured sausages.. J Food Sci.

[r31] Lupo PJ, Lee LJ, Okcu MF, Bondy ML, Scheurer ME (2012). An exploratory case-only analysis of gene-hazardous air pollutant interactions and the risk of childhood medulloblastoma.. Pediatr Blood Cancer.

[r32] Ma Y Harrad S 2015 Spatiotemporal analysis and human exposure assessment on polycyclic aromatic hydrocarbons in indoor air, settled house dust, and diet: a review. Environ Int 84 7 16, doi:10.1016/j.envint.2015.07.006 26197059

[r33] MacDonald TJ (2008). Aggressive infantile embryonal tumors.. J Child Neurol.

[r34] McKean-Cowdin R, Razavi P, Barrington-Trimis J, Baldwin RT, Asgharzadeh S, Cockburn M (2013). Trends in childhood brain tumor incidence, 1973–2009.. J Neurooncol.

[r35] Milne E, Greenop KR, Scott RJ, Ashton LJ, Cohn RJ, de Klerk NH (2013). Parental smoking and risk of childhood brain tumors.. Int J Cancer.

[r36] Naumova YY, Eisenreich SJ, Turpin BJ, Weisel CP, Morandi MT, Colome SD (2002). Polycyclic aromatic hydrocarbons in the indoor and outdoor air of three cities in the U.S.. Environ Sci Technol.

[r37] Ostrom QT de Blank PM Kruchko C Petersen CM Liao P Finlay JL 2015 Alex’s Lemonade Stand Foundation Infant and Childhood Primary Brain and Central Nervous System Tumors Diagnosed in the United States in 2007–2011. Neuro Oncol 16 suppl 10 x1 x35, doi:10.1093/neuonc/nou327 25542864 PMC4277295

[r38] Peters S, Glass DC, Reid A, de Klerk N, Armstrong BK, Kellie S (2013). Parental occupational exposure to engine exhausts and childhood brain tumors.. Int J Cancer.

[r39] Raaschou-Nielsen O, Hertel O, Thomsen BL, Olsen JH (2001). Air pollution from traffic at the residence of children with cancer.. Am J Epidemiol.

[r40] Rajaraman P, Stewart PA, Samet JM, Schwartz BS, Linet MS, Zahm SH (2006). Lead, genetic susceptibility, and risk of adult brain tumors.. Cancer Epidemiol Biomarkers Prev.

[r41] Reynolds P, Von Behren J, Gunier RB, Goldberg DE, Hertz A (2004). Residential exposure to traffic in California and childhood cancer.. Epidemiology.

[r42] Reynolds P, Von Behren J, Gunier RB, Goldberg DE, Hertz A, Smith D (2002). Traffic patterns and childhood cancer incidence rates in California, United States.. Cancer Causes Control.

[r43] Reynolds P Von Behren J Gunier RB Goldberg DE Hertz A Smith DF 2003 Childhood cancer incidence rates and hazardous air pollutants in California: an exploratory analysis. Environ Health Perspect 111 663 668, doi:10.1289/ehp.5986 12676632 PMC1241461

[r44] Rice JM, Wilbourn JD (2000). Tumors of the nervous system in carcinogenic hazard identification.. Toxicol Pathol.

[r45] Ritz B, Wilhelm M, Hoggatt KJ, Ghosh JK (2007). Ambient air pollution and preterm birth in the environment and pregnancy outcomes study at the University of California, Los Angeles.. Am J Epidemiol.

[r46] Rogers HA, Ward JH, Miller S, Lowe J, Coyle B, Grundy RG (2013). The role of the WNT/β-catenin pathway in central nervous system primitive neuroectodermal tumours (CNS PNETs).. Br J Cancer.

[r47] Rosso AL, Hovinga ME, Rorke-Adams LB, Spector LG, Bunin GR, Children’s Oncology Group (2008). A case–control study of childhood brain tumors and fathers’ hobbies: a Children’s Oncology Group study.. Cancer Causes Control.

[r48] Searles Nielsen S McKean-Cowdin R Farin FM Holly EA Preston-Martin S Mueller BA 2010 Childhood brain tumors, residential insecticide exposure, and pesticide metabolism genes. Environ Health Perspect 118 144 149, doi:10.1289/ehp.0901226 20056567 PMC2831959

[r49] Searles Nielsen S, Mueller BA, Preston-Martin S, Farin FM, Holly EA, McKean-Cowdin R (2011). Childhood brain tumors and maternal cured meat consumption in pregnancy: differential effect by glutathione *S*-transferases.. Cancer Epidemiol Biomarkers Prev.

[r50] U.S. Census Bureau (2013). Census 2000 Gateway.. http://www.census.gov/main/www/cen2000.html.

[r51] U.S. Geological Survey (2015). Volatile Organic Compounds in the Nation’s Ground Water and Drinking-Water Supply Wells.. Circular.

[r52] Yost K, Perkins C, Cohen R, Morris C, Wright W (2001). Socioeconomic status and breast cancer incidence in California for different race/ethnic groups.. Cancer Causes Control.

