# Supplementary material for: In Utero and Early-Life Exposure to Ambient Air Toxics and Childhood Brain Tumors: A Population-Based Case–Control Study in California, USA
Source: Environ Health Perspect. 2015 Oct 27;124(7):1093–9. doi: 10.1289/ehp.1408582 (PMC4937846; doi:10.1289/ehp.1408582)
Supplement: (272 KB) PDF [file ehp.1408582.s001.acco.pdf]

Environ Health Perspect
DOI: 10.1289/ehp.1408582

Note to readers with disabilities: *EHP* strives to ensure that all journal content is accessible to all readers. However, some figures and Supplemental Material published in *EHP* articles may not conform to 508 standards due to the complexity of the information being presented. If you need assistance accessing journal content, please contact ehp508@niehs.nih.gov. Our staff will work with you to assess and meet your accessibility needs within 3 working days.

Supplemental Material

In Utero and Early-Life Exposure to Ambient Air Toxics and Childhood Brain Tumors: A Population-Based Case–Control Study in California, USA

Ondine S. von Ehrenstein, Julia E. Heck, Andrew Park, Myles Cockburn, Loraine Escobedo, and Beate Ritz

Table of Contents

Table S1. Air Toxics Measures Entire Pregnancy Averages Among Non-cases 5 miles Distance to Statewide Governmental Air Monitoring Stations, California

Table S2. Adjusted Odds Ratios by Trimesters Exposure to Air Toxics and Primitive Neuroectodermal Tumors in Children by Age 6 Residing within 5-mile Distance to Monitoring Stations at Birth, Birth Years 1990-2007, California

Table S3. Adjusted Odds Ratios by Trimesters Exposure to Air Toxics and Medulloblastoma in Children by Age 6 Years Residing within 5-mile Distance to Monitoring Stations at Birth, Birth Years 1990-2007, California

Table S1. Air Toxics Measures Entire Pregnancy Averages Among Non-cases 5 miles Distance to Statewide Governmental Air Monitoring Stations, California.

**Air Toxic**[a]	**Factor load**[b]	**Mean (SD)**	**IQR**
**Factor 1**			
Aromatic solvents			
Toluene (ppbV)	0.99	2.880 (1.772)	2.196
Ortho-Xylene (ppbV)	0.99	0.495 (0.334)	0.388
Ethyl Benzene (ppbV)	0.98	0.378 (0.189)	0.178
1,3-Butadiene (ppbV)	0.97	0.297 (0.179)	0.257
Benzene (ppbV)	0.97	1.295 (0.862)	1.216
Chlorinated solvents			
Perchloroethylene (ppbV)	0.94	0.203 (0.202)	0.231
Tricloroethylene (ppbV)	0.68	0.080 (0.117)	0.054
Methylene Chloride (ppbV)	0.92	0.740 (0.595)	0.453
Other			
Hexavalent Chromium (ng/m3)	0.90	0.193 (0.112)	0.134
Lead (ng/m3)	0.89	22.99 (18.68)	20.05
Styrene (ppbV))	0.87	0.163 (0.124)	0.137
Acetaldehyde (ppbV)	0.86	1.417 (0.686)	0.900
Selenium (ng/m3)	0.69	1.558 (0.701)	0.732
**Factor 2**			
PAHs[c] (ng/m3)	0.99	1.437 (1.060)	1.049
Benzo(k)fluoranthene (ng/m3)	0.99	0.103 (0.094)	0.077
Benzo(b)fluoranthene (ng/m3)	0.99	0.254 (0.221)	0.192
Indeno(1,2,3-cd)pyrene (ng/m3)	0.99	0.309 (0.238)	0.233
Benzo(a)pyrene (ng/m3)	0.98	0.199 (0.210)	0.157
Dibenz(a,h)anthracene (ng/m3)	0.94	0.035 (0.034)	0.015
Benzo(g,h,i)perylene (ng/m3)	0.86	0.559 (0.348)	0.448
Other			
Chloroform (ppbV)	0.84	0.036 (0.014)	0.017
Ortho-Dichlorobenzene (ppbV)	0.83	0.113 (0.040)	0.076
Para-Dichlorobenzene (ppbV)	0.81	0.147 (0.038)	0.039
Formaldehyde (ppbV)	0.71	2.867 (1.197)	1.334
**Not loading**			
Chromium (ng/m3)	--	5.302 (2.731)	3.206
Nickel (ng/m3)	--	5.072 (2.263)	3.196

[a] included are substances with at least 20 exposed cases.

[b]Factor loadings for given factors absolute values: >0.60; Varimax Rotation Factor Pattern.

[c]PAH: Includes sum of average concentrations of six hydrocarbons: benzo[a]pyrene, benzo[b]flouranthene, benzo[ghi]perylene, benzo[k]flouranthene, dibenz[a, h]anthracene, and indeno[1, 2, 3-c,d]pyrene.

Table S2. Adjusted[a] Odds Ratios by Trimesters Exposure to Air Toxics and Primitive Neuroectodermal Tumors in Children by Age 6 Residing within 5-mile Distance to Monitoring Stations at Birth, Birth Years 1990-2007, California.

		Trimester 1		Trimester 2		Trimester 3	
**Air Toxic**	**IQR**	**Cases**	**OR[a] 95% CI**	**Cases**	**OR[a] 95%CI**	**Cases**	**OR[a] 95% CI**
**Factor 1**							
Aromatic solvents							
Toluene (ppbV)	2.196	37/24149	1.63 (1.23, 2.16)	37/24149	1.43 (1.06, 1.92)	37/24079	1.50 (1.10, 2.05)
Ortho-Xylene (ppbV)	0.388	37/24033	1.45 (1.10, 1.92)	37/24033	1.54 (1.18, 2.01)	37/23962	1.19 (0.85, 1.65)
Ethyl Benzene (ppbV)	0.178	35/23267	1.27 (1.03, 1.56)	35/23267	1.29 (1.07, 1.55)	35/23200	1.09 (0.83, 1.42)
1,3-Butadiene (ppbV)	0.257	38/27189	1.50 (1.08, 2.08)	38/27189	1.53 (1.11, 2.12)	38/27121	1.13 (0.78, 1.65)
Benzene (ppbV)	1.216	38/27199	1.59 (1.05, 2.42)	38/27199	1.73 (1.12, 2.67)	38/27131	1.15 (0.70, 1.89)
Chlorinated solvents							
Perchloroethylene (ppbV)	0.231	36/25061	1.14 (0.99, 1.31)	36/25061	1.19 (1.05, 1.34)	36/24996	1.16 (0.99, 1.36)
Tricloroethylene (ppbV)	0.054	36/25168	1.09 (1.02, 1.16)	36/25168	1.05 (0.96, 1.15)	36/25103	1.12 (1.07, 1.18)
Methylene Chloride (ppbV)	0.453	34/25412	1.03 (0.92, 1.16)	34/25412	1.04 (0.95, 1.15)	34/25342	1.06 (0.94, 1.19)
Other							
Hexavalent Chromium (ng/m3)	0.134	26/16944	1.05 (0.86, 1.28)	26/16944	1.10 (0.99, 1.22)	26/16894	0.99 (0.67, 1.46)
Lead (ng/m3)	20.048	26/19765	1.23 (0.85, 1.79)	26/19765	1.38 (0.98, 1.97)	26/19713	1.10 (0.73, 1.64)
Styrene (ppbV))	0.137	29/20001	1.31 (0.99, 1.73)	29/20001	1.24 (0.94, 1.64)	29/19938	0.99 (0.69, 1.43)
Acetaldehyde (ppbV)	0.900	34/25361	1.95 (1.37, 2.76)	34/25361	1.46 (1.01, 2.09)	34/25296	1.62 (1.14, 2.31)
Selenium (ng/m3)	0.732	25/18999	1.23 (0.98, 1.56)	25/18999	1.40 (1.17, 1.67)	25/18949	1.08 (0.80, 1.45)
**Factor 2**							
PAHs[b] (ng/m3)	1.049	29/21368	0.99 (0.79, 1.24)	29/21368	1.05 (0.90, 1.22)	29/21309	0.99 (0.77, 1.26)
Benzo(k)fluoranthene (ng/m3)	0.077	30/22416	1.02 (0.86, 1.20)	30/22416	1.01 (0.86, 1.18)	30/22355	0.92 (0.72, 1.18)
Benzo(b)fluoranthene (ng/m3)	0.192	30/22416	1.05 (0.90, 1.22)	30/22416	1.01 (0.86, 1.19)	30/22355	0.92 (0.71, 1.19)
Indeno(1,2,3-cd)pyrene (ng/m3)	0.233	29/21368	0.98 (0.78, 1.23)	29/21368	1.04 (0.89, 1.21)	29/21309	0.99 (0.77, 1.27)
Benzo(a)pyrene (ng/m3)	0.157	30/22416	1.03 (0.90, 1.18)	30/22416	0.97 (0.81, 1.17)	30/22355	0.91 (0.71, 1.16)
Dibenz(a,h)anthracene (ng/m3)	0.015	29/21368	0.94 (0.79, 1.13)	29/21368	0.94 (0.76, 1.16)	29/21309	0.87 (0.64, 1.19)
Benzo(g,h,i)perylene (ng/m3)	0.448	29/21368	1.10 (0.83, 1.44)	29/21368	1.21 (0.97, 1.49)	29/21309	1.05 (0.79, 1.39)
Other							
Chloroform (ppbV)	0.017	37/25534	1.28 (1.01, 1.62)	37/25534	1.29 (1.01, 1.64)	37/25468	1.25 (0.96, 1.62)
Ortho-Dichlorobenzene (ppbV)	0.076	32/21053	1.23 (0.76, 2.01)	32/21053	1.51 (0.98, 2.34)	32/20991	0.99 (0.57, 1.70)
Para-Dichloro-benzene (ppbV)	0.039	32/21121	1.11 (0.95, 1.31)	32/21121	1.13 (0.97, 1.32)	32/21059	1.07 (0.88, 1.30)
Formaldehyde (ppbV)	1.334	34/25361	1.15 (1.01, 1.31)	34/25361	1.06 (0.77, 1.47)	34/25296	1.34 (1.01, 1.77)
**Not loading**							
Chromium (ng/m3)	3.206	26/19867	1.20 (0.88, 1.64)	26/19867	1.22 (0.92, 1.63)	26/19815	1.09 (0.77, 1.54)
Nickel (ng/m3)	3.196	26/19889	1.28 (0.84, 1.93)	26/19889	1.14 (0.72, 1.80)	26/19837	0.97 (0.59, 1.57)

[a] Adjusted for birth year. [+]Adjusted for: birth year, maternal race/ethnicity, maternal age and education, place of birth mother (US vs. non US);

[b]PAH: Includes sum of average concentrations of six hydrocarbons: benzo[a]pyrene, benzo[b]flouranthene, benzo[ghi]perylene, benzo[k]flouranthene, dibenz[a, h]anthracene, and indeno[1, 2, 3-c,d]pyrene.

Table S3. Adjusted[a] Odds Ratios by Trimesters Exposure to Air Toxics and Medulloblastoma in Children by Age 6 Years Residing within 5-mile Distance to Monitoring Stations at Birth, Birth Years 1990-2007, California.

		Trimester 1		Trimester 2		Trimester 3	
**Air Toxic**	**IQR**	**Cases**	**OR[a] 95% CI**	**Cases**	**OR[a] 95%CI**	**Cases**	**OR[a] 95% CI**
**Factor 1**							
Aromatic solvents							
Toluene (ppbV)	2.196	27/24149	0.96 (0.6, 1.54)	27/24149	0.87 (0.54, 1.41)	27/24079	0.50 (0.26, 0.97)
Ortho-Xylene (ppbV)	0.388	27/24033	1.04 (0.69, 1.58)	27/24033	0.94 (0.61, 1.45)	27/23962	0.57 (0.31, 1.05)
Ethyl Benzene (ppbV)	0.178	24/23267	0.79 (0.50, 1.26)	24/23267	0.81 (0.53, 1.26)	24/23200	0.53 (0.27, 1.02)
1,3-Butadiene (ppbV)	0.257	30/27189	1.00 (0.63, 1.57)	30/27189	1.07 (0.69, 1.65)	30/27121	0.59 (0.32, 1.07)
Benzene (ppbV)	1.216	30/27199	1.19 (0.70, 2.01)	30/27199	1.08 (0.61, 1.89)	30/27131	0.46 (0.22, 0.96)
Chlorinated solvents							
Perchloroethylene (ppbV)	0.231	28/25061	0.76 (0.43, 1.34)	28/25061	0.50 (0.24, 1.05)	28/24996	0.44 (0.19, 0.98)
Tricloroethylene (ppbV)	0.054	28/25168	1.01 (0.87, 1.17)	28/25168	0.89 (0.67, 1.19)	28/25103	0.91 (0.69, 1.20)
Methylene Chloride (ppbV)	0.453	28/25412	0.76 (0.48, 1.20)	28/25412	0.69 (0.41, 1.15)	28/25342	0.87 (0.59, 1.30)
Other							
Hexavalent Chromium (ng/m3)	0.134	20/16944	0.31 (0.1, 1.02)	20/16944	0.38 (0.12, 1.17)	20/16894	0.85 (0.39, 1.86)
Lead (ng/m3)	20.048	21/19765	1.43 (0.94, 2.17)	21/19765	0.67 (0.34, 1.36)	21/19713	0.64 (0.30, 1.33)
Styrene (ppbV))	0.137	25/20001	1.07 (0.73, 1.58)	25/20001	1.05 (0.73, 1.52)	25/19938	0.66 (0.36, 1.20)
Acetaldehyde (ppbV)	0.900	27/25361	1.01 (0.63, 1.61)	27/25361	0.91 (0.55, 1.48)	27/25296	0.76 (0.45, 1.28)
Selenium (ng/m3)	0.732	20/18999	1.23 (0.92, 1.66)	20/18999	1.03 (0.71, 1.49)	20/18949	0.70 (0.37, 1.32)
**Factor 2**							
PAHs[b] (ng/m3)	1.049	27/21368	1.13 (1.01, 1.26)	27/21368	1.10 (0.99, 1.22)	27/21309	1.04 (0.84, 1.29)
Benzo(k)fluoranthene (ng/m3)	0.077	28/22416	1.10 (1.01, 1.20)	28/22416	1.07 (0.98, 1.16)	28/22355	1.02 (0.87, 1.18)
Benzo(b)fluoranthene (ng/m3)	0.192	28/22416	1.12 (1.03, 1.22)	28/22416	1.08 (0.98, 1.18)	28/22355	1.02 (0.86, 1.20)
Indeno(1,2,3-cd)pyrene (ng/m3)	0.233	27/21368	1.12 (1.01, 1.24)	27/21368	1.10 (0.99, 1.21)	27/21309	1.05 (0.85, 1.31)
Benzo(a)pyrene (ng/m3)	0.157	28/22416	1.09 (1.01, 1.17)	28/22416	1.06 (0.99, 1.14)	28/22355	1.01 (0.88, 1.16)
Dibenz(a,h)anthracene (ng/m3)	0.015	27/21368	1.02 (0.98, 1.06)	27/21368	1.02 (0.99, 1.05)	27/21309	1.08 (0.94, 1.24)
Benzo(g,h,i)perylene (ng/m3)	0.448	27/21368	1.28 (1.02, 1.62)	27/21368	1.18 (0.95, 1.47)	27/21309	0.98 (0.71, 1.34)
Other							
Chloroform (ppbV)	0.017	28/25534	1.10 (0.78, 1.53)	28/25534	0.85 (0.55, 1.3)	28/25468	0.61 (0.37, 1.01)
Ortho-Dichlorobenzene (ppbV)	0.076	23/21053	0.98 (0.49, 1.95)	23/21053	0.96 (0.49, 1.89)	23/20991	0.34 (0.13, 0.90)
Para-Dichloro-benzene (ppbV)	0.039	23/21121	1.00 (0.76, 1.32)	23/21121	0.90 (0.64, 1.27)	23/21059	0.99 (0.75, 1.32)
Formaldehyde (ppbV)	1.334	27/25361	0.92 (0.62, 1.38)	27/25361	0.82 (0.54, 1.27)	27/25296	0.84 (0.56, 1.27)
**Not loading**							
Chromium (ng/m3)	3.206	21/19867	1.09 (0.74, 1.61)	21/19867	0.80 (0.46, 1.39)	21/19815	0.66 (0.35, 1.22)
Nickel (ng/m3)	3.196	21/19889	1.01 (0.60, 1.70)	21/19889	0.70 (0.37, 1.30)	21/19837	0.58 (0.30, 1.14)

[a] Adjusted for birth year. [+]Adjusted for: birth year, maternal race/ethnicity, maternal age and education, place of birth mother (US vs. non US).

[b]PAH: Includes sum of average concentrations of six hydrocarbons: benzo[a]pyrene, benzo[b]flouranthene, benzo[ghi]perylene, benzo[k]flouranthene, dibenz[a, h]anthracene, and indeno[1, 2, 3-c,d]pyrene.
